# Genes responding to Canakinumab therapy in SJIA are -inversely - disregulated in adult onset Still's disease

**DOI:** 10.1186/1546-0096-13-S1-P4

**Published:** 2015-09-28

**Authors:** A Brachat, E Feist, F Behrens, N Blank, NR Nirmala, C Specker, M Witt, J Zernicke, A Martini, G Junge

**Affiliations:** 1 Novarts Institutes for Biomedical Research, Basel, Switzerland; 2 Charité - University Hospital Berlin, Berlin, Germany; 3 Klinikum Johann Wolfgang Goethe - Universität, Frankfurt, Germany; 4 University of Heidelberg, Heidelberg, Germany; 5 Novartis Institutes of Biomedical Research, Cambridge, USA; 6 Kliniken Essen Süd, Essen, Germany; 7 University of Munich, Munich, Germany; 8 G Gaslini Institute, Genova, Italy; 9 Novartis Pharma AG, Basel, Switzerland

## Introduction

Adult-onset Still's disease (AOSD) is a rare auto-inflammatory disorder resembling a similar pediatric syndrome known as systemic juvenile idiopathic arthritis (SJIA).[^1^] The superimposable systemic and clinical features in SJIA and AOSD suggest that both clinical phenotypes represent a disease continuum with a pediatric (SJIA) and more adult-onset (AOSD).[^2^] Analyses of gene expression profiles may be useful not only for disease classification, diagnosis, and prognosis, but also to identify disease specific treatment effects that counteract the underlying pathological mechanisms. Here, we address the question: How do genes that respond to canakinumab treatment in SJIA patients[^3^] behave in AOSD patients with active disease relative to healthy controls and prior to IL-1 targeting therapy?

## Objectives

To determine how genes that respond to IL-1β blockade with canakinumab in SJIA patients behave in AOSD patients relative to healthy controls.

## Patients and methods

SJIA gene expression profiles pre- and post canakinumab treatment were compared with AOSD patients relative to healthy subjects using Affymetrix U133Plus2 DNA microarrays.

## Results

Consistently, all genes down-regulated in SJIA following canakinumab treatment were upregulated in a majority of AOSD patients with active disease relative to healthy subjects and prior to canakinumab treatment. A few of the AOSD patients resembled healthy subjects. Comparison of the gene expression patterns to neutrophil counts suggested that elevated neutrophil numbers were closely correlated to the up-regulation of IL-1 associated gene expression.

## Conclusions

Results are consistent with and further support the concept of a Still's disease continuum that presents as pediatric/juvenile SJIA or adult-onset Still's disease. Moreover, they suggest that AOSD is an IL-1 driven condition that is also mechanistically similar to SJIA and that the observed canakinumab response signature is likely to show a comparable treatment response to IL-1β blockade in AOSD.
